# Positron range in combination with point-spread-function correction: an evaluation of different implementations for [124I]-PET imaging

**DOI:** 10.1186/s40658-022-00482-y

**Published:** 2022-08-19

**Authors:** Hunor Kertész, Maurizio Conti, Vladimir Panin, Jorge Cabello, Deepak Bharkhada, Thomas Beyer, Laszlo Papp, Walter Jentzen, Jacobo Cal-Gonzalez, Joaquín L. Herraiz, Alejandro López-Montes, Ivo Rausch

**Affiliations:** 1 QIMP Team, Center for Medical Physics and Biomedical Engineering, Medical University of Vienna, Währinger Gürtel 18-20, 1090 Vienna, Austria; 2 Siemens Medical Solutions USA, Inc., Knoxville, TN USA; 3 Clinic for Nuclear Medicine, University Hospital Essen, Essen, Germany; 4 Ion Beam Applications, Protontherapy Center Quironsalud, Madrid, Spain; 5 Nuclear Physics Group and IPARCOS, Faculty of Physical Sciences, University Complutense of Madrid, Madrid, Spain; 6 Health Research Institute of the Hospital Clínico San Carlos, Madrid, Spain

**Keywords:** PET, Positron range correction, PRC, Image reconstruction

## Abstract

**Aim:**

To evaluate the effect of combining positron range correction (PRC) with point-spread-function (PSF) correction and to compare different methods of implementation into iterative image reconstruction for ^124^I-PET imaging.

**Materials and methods:**

Uniform PR blurring kernels of ^124^I were generated using the GATE (GEANT4) framework in various material environments (lung, water, and bone) and matched to a 3D matrix. The kernels size was set to 11 × 11 × 11 based on the maximum PR in water and the voxel size of the PET system. PET image reconstruction was performed using the standard OSEM algorithm, OSEM with PRC implemented before the forward projection (OSEM+PRC simplified) and OSEM with PRC implemented in both forward- and back-projection steps (full implementation) (OSEM+PRC). Reconstructions were repeated with resolution recovery, point-spread function (PSF) included. The methods and kernel variation were validated using different phantoms filled with ^124^I acquired on a Siemens mCT PET/CT system. The data was evaluated for contrast recovery and image noise.

**Results:**

Contrast recovery improved by 2–10% and 4–37% with OSEM+PRC simplified and OSEM+PRC, respectively, depending on the sphere size of the NEMA IQ phantom. Including PSF in the reconstructions further improved contrast by 4–19% and 3–16% with the PSF+PRC simplified and PSF+PRC, respectively. The benefit of PRC was more pronounced within low-density material. OSEM-PRC and OSEM-PSF as well as OSEM-PSF+PRC in its full- and simplified implementation showed comparable noise and convergence. OSEM-PRC simplified showed comparably faster convergence but at the cost of increased image noise.

**Conclusions:**

The combination of the PSF and PRC leads to increased contrast recovery with reduced image noise compared to stand-alone PSF or PRC reconstruction. For OSEM-PRC reconstructions, a full implementation in the reconstruction is necessary to handle image noise. For the combination of PRC with PSF, a simplified PRC implementation can be used to reduce reconstruction times.

**Supplementary Information:**

The online version contains supplementary material available at 10.1186/s40658-022-00482-y.

## Background

Positron emission tomography (PET) is based on the detection of photon radiation arising from the annihilation of positrons originating from the PET tracers with electrons within the patient [1]. To obtain a 3D representation of the tracer distribution, image reconstruction is necessary. This is done today using iterative reconstruction algorithms where the detector signals are estimated from a guess of the tracer distribution using a mathematical model of the system (system matrix) and compared to the actual measured signal in projection space. Standard reconstruction algorithms, such as OSEM, typically use simple geometric assumptions to model the system matrix [2]. However, to optimize spatial resolution a more realistic model of the system accounting for all physical processes is needed. A realistic system response is, for example, derived from point source measurements [3]. The implementation of such a realistic detector response function into the projectors of the iterative image reconstruction process is called resolution recovery or point-spread-function (PSF) correction. The benefit of including PSF in the image reconstruction by means of reduced image noise and increased contrast was shown by various studies [4–7].

However, besides the imaging systems properties, spatial resolution in PET is further limited by positron range (PR), the distance traveled by the positron from the emission position to the annihilation position [8, 9]. For the most commonly used PET isotope (^18^F), median PR in soft tissue or water is of the order of 0.4 mm and thus [10] can be practically ignored in clinical PET studies. However, positron emitters with high positron energies such as ^68^Ga and ^124^I, which are increasingly applied in the diagnosis of prostate cancer [11] or used for therapy planning in patients with thyroid cancer [12], respectively, present with average PRs of ~ 10 mm in soft tissues which lead to substantial degradation of spatial resolution and image quality [13].

Positron range effects can be corrected with a similar implementation to the one used to incorporate PSF into the image reconstruction algorithm. This is done by adding the PR-induced blurring to the forward projector, for example, by convolving the current image guess with the PR-based blurring kernels before projecting the current image guess into projection space. In addition, the PR blurring has to be taken into account in the back-projection step within an iterative reconstruction since the back-projection is a transpose operation of the forward projection [14]. The main challenges of PRC are deriving adequate PR kernels and extensive computational demand due to the use of non-isotropic and non-shift-invariant PR kernels. Monte Carlo simulations are usually used for defining the ground truth of tissue-dependent PR kernels [10].

However, due to the computational challenges, PR kernels are generally based on simply using isotropic PR kernels for different tissue types [15–17]. Only a limited number of studies used spatially variant and tissue-dependent estimations of PR kernels including effects on tissue borders [15, 18]. These studies suggest implementing the PR blurring kernels only prior to the forward projection step in standard iterative reconstructions as an image blur to reduce the computational demand [15, 19]. Only one recent study showed the value of using a full implementation of the PRC into an OSEM algorithm [14].

Therefore, our study aimed to evaluate the effect of combined PSF correction and PRC within the reconstruction on image contrast and convergence. Further, the influence of using simplified PR correction implementation methods limiting computational demand in combination with PSF was assessed.

## Methods

### Positron range kernel calculation

The uniform positron range distribution of ^124^I was simulated using GATE 9.0 (GEANT4 10.06.p02). The simulation setup consisted of a point source with a radius of 5 nm centered in a uniform phantom with a radius of 30 cm. For the phantom material, three settings were simulated bone material (mass density 1.92 g/cm^3^), water (1.00 g/cm^3^), and lung equivalent material (0.26 g/cm^3^). The predefined “empenelope” physics list was used. The initial activity of ^124^I was 10 MBq and the emission and annihilation coordinates were recorded for 20 M annihilation events. From the recorded data, the 3D PR distribution was generated by mapping the distribution to a 3D matrix. The size of the kernel (11 × 11 × 11 voxels) was chosen based on the voxel size of the Siemens mCT PET/CT system (2 × 2 × 2 mm^3^) (Siemens Medical Solutions Inc., Knoxville, TN, USA) [20] and the maximum PR in water for ^124^I (~ 10 mm), since the use of larger kernels is leading to artifacts [14].

For each voxel of the investigated object, spatially variant and tissue-dependent PR kernels based on the underlying material distribution were created as described before [14]. In short, the attenuation map was segmented into a material map containing lung, soft tissue, and bone. Regions containing air were treated as lung material. Then, for every voxel, PR kernels were generated by combining pre-calculated uniform PR kernels for lung, water, and bone from GATE Monte Carlo simulations.

### Positron range correction implementations

The PR correction was implemented into the vendor-based software in combination with OSEM and PSF algorithms as an additional PR-dependent PSF applied in image space. This was done as full implementation and, in addition, in a simplified version applying the PR-dependent blurring only before the forward projector of the reconstruction algorithm, as suggested by [19] to reduce computational demand.

The standard OSEM algorithm taken as the basis can be written as:

1 $$f_{j}^{n + 1} = \frac{{f_{j}^{n} }}{{\sum\nolimits_{{i^{\prime} \in S^{n} }} {a_{i^{\prime}j} } }}\sum_{{i \in S^{n} }} a_{ij} \frac{{m_{i} }}{{\sum_{k} a_{ik} f_{k}^{n} }}$$

where *f*_*j*_^*n*+*1*^ is the next image estimate of voxel *j* based on the current image estimate *f*_*k*_^*n*^. The measured projection data is *m*_*i*_*.* The system matrix describing the probability that the emission from voxel *j* will be detected along the line of response (LOR) *i* is given by *A*_*ij*_ = *(a*_*ij*_*)*_*IxJ*_. Only a subset *S*^*n*^ of the data was used in each update. Of note: for simplification, the random and scatter are not included in this description. The system matrix can be factorized to account for the finite resolution effects, in our case the positron range effect by matrix *H* = *(h*_*j’j*_*)*_*JxJ*_, a matrix *X* = *(x*_*ij*_*)*_*IxJ*_ expressing the intersection length and a matrix *W* = *(w*_*ii*_*)*_*IxI*_ describing the photon attenuation and geometrical sensitivity variations. Thus, the system matrix can be written as:

2 $$A =WXH$$

Combining Eq. (1) and Eq. (2), the OSEM algorithm with PRC correction can be written as [14]:

3 $${f}_{j}^{n+1}=\frac{{f}_{j}^{n}}{{\sum }_{b}{h}_{bj}{\sum }_{i\in {S}^{n}}{w}_{ii}{x}_{ib}}{\sum }_{b}{h}_{bj}{\sum }_{i\in {S}^{n}}{x}_{ib}\frac{{m}_{i}}{{\sum }_{p}{x}_{ip}{\sum }_{v}{h}_{pv}{f}_{v}^{n}}$$

This method is referred to as “PRC” in this work. Since PRC is computationally highly expensive, a simplified version of the PRC implementation was suggested in previous studies [18, 21], where the PR kernels are applied to the current image estimate as a convolution in image space before the forward projection. In this way, Eq. (1) can be written as:

4 $$f_{j}^{n + 1} = \frac{{f_{j}^{n} }}{{\sum\nolimits_{{i^{\prime} \in S^{n} }} {a_{i^{\prime}j} } }}\sum_{{i \in S^{n} }} a_{ij} \frac{{m_{i} }}{{\sum_{k} a_{ik} f_{k}^{{n^{ \sim } }} }}$$

where $f_{k}^{{n^{ \sim } }}$ is the current image estimate blurred with the spatially variant and tissue-dependent PR kernel ($\rho$) calculated for the given voxel [19]:

5 $$f_{k}^{{n^{ \sim } }} = f \otimes \rho = \frac{{\sum_{h} f_{k} - \rho_{h} }}{{\mathop \sum \nolimits_{h}^{{}} \rho_{h} }}$$

This method is referred to as “PRC simplified” in this study. Both PRC methods (PRC and PRC simplified) together with the generated PR kernels were implemented into the Siemens e7tools (Siemens Medical Solutions USA, Inc., Knoxville, TN, USA) image reconstruction framework using MATLAB R2019a (MathWorks Inc, USA). The image reconstructions with both PRC implementations were performed with both OSEM and PSF corrected OSEM (PSF): OSEM algorithm with PR implemented in the forward projection only (OSEM+PRC simplified), OSEM with PRC implemented in both forward- and back-projection (OSEM+PRC). The same naming convention was used for PSF reconstructions (PSF+PRC simplified and PSF+PRC).

All reconstructions were done with 1–10 iterations and 12 subsets, except the reconstruction for which PSF was combined with PRC in the back-projection (PSF+PRC). In this case, image reconstructions were performed with 1–20 iterations and 12 subsets. The image size was 400 × 400 x 109 voxels, with a voxel size of 2 × 2 × 2 mm^3^. All emission data was corrected for attenuation, normalization, scatter, and randoms. The time-of-flight (TOF) information was included and no post-reconstruction filters were applied.

### Comparison of the PRC implementations

The PRC implementations were tested and compared by means of contrast, noise, and convergence properties using the standard “NEMA Image Quality (IQ)” phantom, a modified NEMQ IQ phantom with changed hot spheres referred to as “Small-tumor” phantom and a “Bone–lung” phantom. Of note, the NEMA IQ phantom simulates hot lesions embedded in soft tissue (water) only (Additional file 1: Fig. S1a). The Small-tumor phantom is a specially-modified phantom using the housing of the NEMA IQ phantom, with fillable spheres with diameters of 3.7, 4.8, 6.5, 7.7, 8.9, and 9.7 mm (Additional file 1: Fig. S1b). The dedicated Bone–lung phantom simulates hot lesions embedded in lung and bone mimicking material, and thus, allows a comparison of the PRCs in a tissue-dependent environment (Additional file 1: Fig. S1c). All phantom acquisitions were performed at the University Clinic Essen, Germany, using the Siemens mCT PET/CT system [20].

#### NEMA IQ

In the NEMA IQ phantom, all the spheres (diameter of 10, 13, 17, 22, 28, and 37 mm) were filled with a ^124^I activity concentration of 30 kBq/ml, and the background region was filled with an activity concentration of 6 kBq/ml. The emission acquisition time was 60 min. The phantom was positioned with the centers of the spheres in the center of FOV. For the evaluation, 12 volume-of-interests (VOIs) were used in the background region (each with a diameter of 37 mm) and 6 individual VOIs covering every sphere (with the diameter corresponding to the sphere size) (Additional file 1: Fig. S1d). For placing the background VOIs, the center of the FOV slice was selected, where all the spheres are centered as well. The reconstructed images were evaluated for contrast recovery calculated as:

6 $${\text{Contrast}}\; {\text{recovery}} = \frac{{\frac{{{\text{MEAN}}_{{{\text{signal}}}} }}{{{\text{MEAN}}_{{{\text{background}}}} }}}}{{{\text{Activity }}\;{\text{ratio}}}}$$

where the mean value in the background was calculated as the mean overall VOIs.

Image noise was defined in percentage following the guidelines of EFOMP [22]:

7 $${\text{Noise}} = \frac{{{\text{STDEV}}_{{{\text{background}}}} }}{{{\text{MEAN}}_{{{\text{background}}}} }}*100$$

The convergence of the investigated reconstruction algorithms in combination with the various PRC implementations was assessed by plotting the contrast recovery vs. noise and how fast contrast recovery and noise is changing with each iteration [14, 23]. For a direct comparison of the reconstructed images, the reconstruction settings were selected to match a 10% background noise as defined as a clinical acceptable noise level as gained from phantom scans [22].

#### Small-tumor

The Small-tumor phantom was filled with a ^124^I activity concentration of 25 kBq/ml in the hot lesions and a background activity of 1.2 kBq/ml leading to a signal-to-noise ratio of 20:1. The acquisition time was 30 min [7]. The reconstructed images were evaluated for contrast recovery (Eq. 5) and image noise (Eq. 6) by defining 10 VOIs in the background region (diameter of 20 mm) and individual VOIs for every sphere (with the diameter corresponding to the sphere size) (Additional file 1: Fig. S1e).

#### Bone–lung

The Bone–lung phantom was composed of 3 different cylinders (each with a diameter of 50 mm) made of different materials (lung with -800 HU, bone with 500 HU, and 1000 HU) that are housed within the standard NEMA IQ phantom [24]. Within every cold cylinder, two fillable spheres were inserted, one with a diameter of 8.5 mm and one with 19.4 mm. The spheres were filled with a ^124^I activity concentration of 30 kBq/ml and the background with 6 kBq/ml. Similar to the NEMA IQ evaluations, 6 background VOIs (diameter of 37 mm) and VOIs covering the hot spheres (diameter of 8.5 and 19.4 mm) were defined (Additional file 1: Fig. S1f). The acquisition time was 60 min, and the reconstructed images were evaluated for contrast recovery (Eq. 5) and noise (Eq. 6).

## Results

### NEMA IQ phantom

Substantial differences in convergence, image contrast, and noise properties were noticed between the different PRC implementations.

**Fig. 1:**
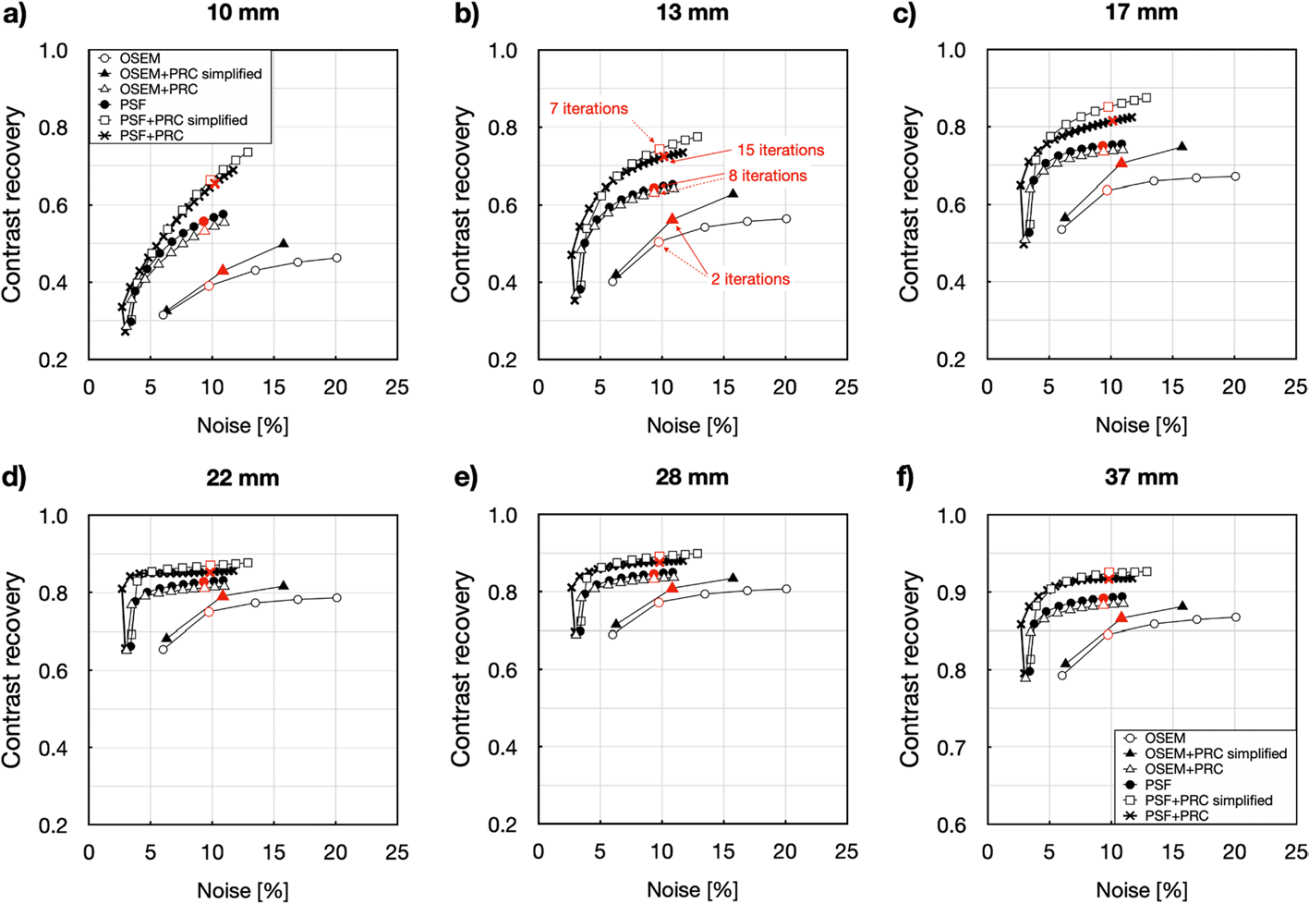
Noise versus contrast recovery for the NEMA IQ phantom: (**a**) 10 mm, (**b**) 13 mm, (**c**) 17 mm, (**d**) 22 mm, (**e**) 28 mm and (**f**) 37 mm spheres filled with ^124^I and reconstructed with OSEM (1–5 iterations), OSEM+PRC simplified (1–3 iterations), OSEM+PRC (1–10 iterations), PSF (1–10 iterations), PSF+PRC simplified (1–10 iterations), and PSF+PRC (1–20 iterations). Every point corresponds to one iteration (all reconstructions done with 12 subsets). The behavior of the OSEM+PRC is similar to the stand-alone PSF reconstructions. The selected number of iterations for the comparison of the images is marked with red

In general, three different convergence patterns were observed that can be described as follows: OSEM+PRC simplified had similar convergence as OSEM with higher achievable contrast but also noise levels (Fig. 1). In both cases, 2 iterations and 12 subsets resulted in the background noise of approximately 10%.

OSEM+PRC and PSF showed similar convergence, contrast, and noise level with improved contrast and noise properties, albeit with a slower enhancement of noise (the 10% noise level was reached with 8 iterations and 12 subsets) compared to OSEM and OSEM+PRC simplified (Fig. 1). PRC in combination with PSF resulted in similar convergence and contract recovery for the full and simplified implementation; however, convergence was notably slower by means of gain in contrast per iteration for PSF+PRC compared to PSF+PRC simplified (Fig. 1).

**Fig. 2:**
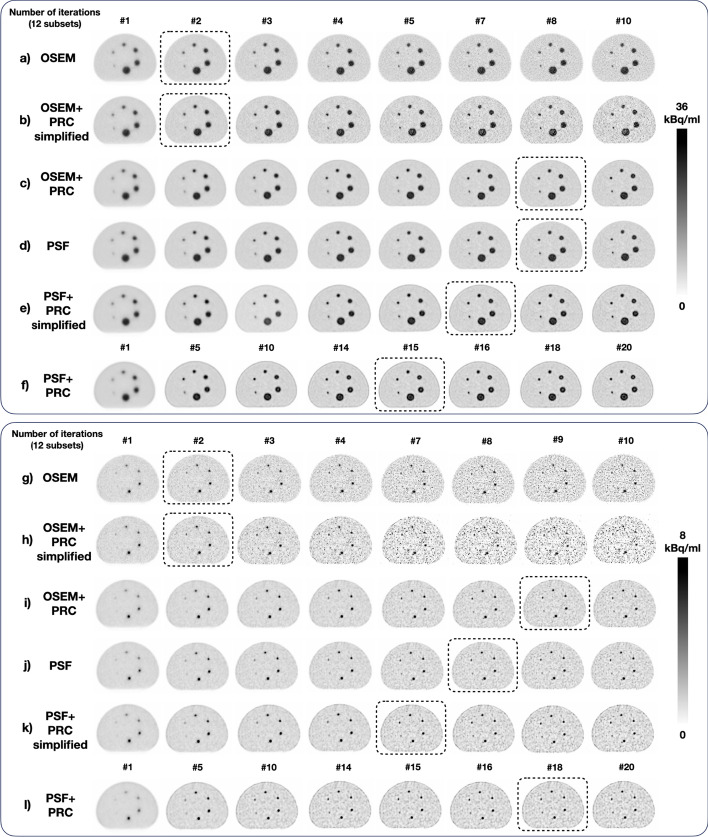
Comparison of the central axial slice of the reconstructed (top) NEMA IQ phantom using: (**a**) OSEM; (**b**) OSEM+PRC simplified; (**c**) OSEM+PRC; (**d**) PSF; (**e**) PSF+PRC simplified; and (**f**) PSF+PRC. The images with similar noise levels measured in the background are marked with dashed boxes. These noise levels were achieved using 2 iterations for OSEM and OSEM+PRC simplified, 8 iterations from OSEM+PRC and PSF, 7 iterations for PSF+PSF simplified, and 15 iterations for PSF+PRC. All reconstructions were with 12 subsets. The OSEM+PRC is producing similar images then the stand-alone PSF; however, more pronounced Gibbs artifacts are produced. (bottom) Small-tumor phantom using: (**g**) OSEM; (**h**) OSEM+PRC simplified; (**i**) OSEM+PRC; (**j**) PSF; (**k**) PSF+PRC simplified, and l) PSF+PRC. The images with similar noise levels measured in the background (~ 33%) are marked with dashed boxes. These noise levels were achieved using 2 iterations for OSEM and OSEM+PRC simplified, 9 iterations from OSEM+PRC, 8 iterations for PSF, 7 iterations for PSF+PSF simplified, and 18 iterations for PSF+PRC All reconstructions were with 12 subsets

**Fig. 3:**
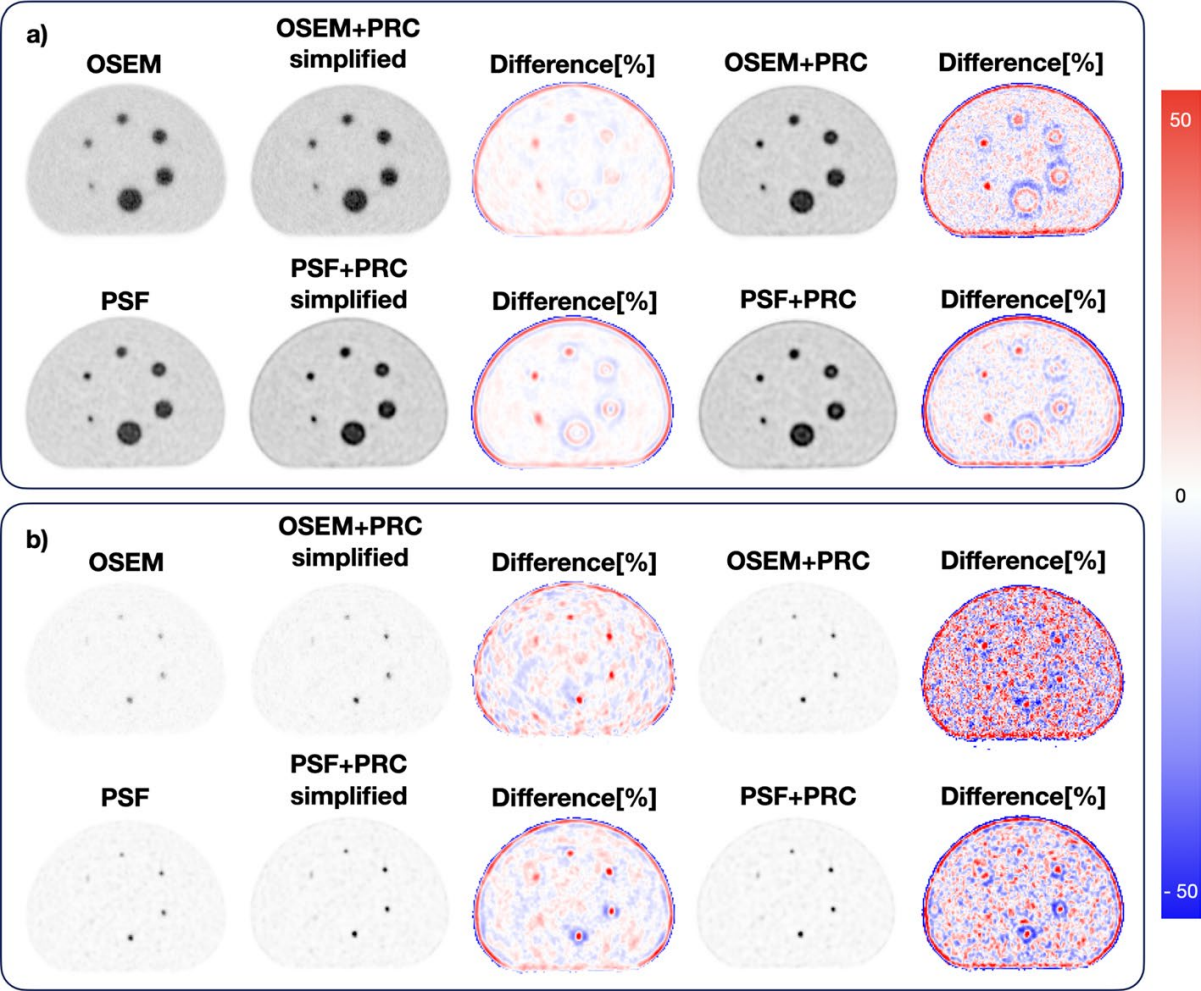
Comparison of the reconstructed (**a**) NEMA Image Quality phantom with OSEM (2 iterations and 12 subsets), OSEM+PRC simplified (2 iterations and 12 subsets), OSEM+PRC (8 iterations and 12 subsets), PSF (8 iterations and 12 subsets), PSF+PRC simplified (7 iterations and 12 subsets), and PSF+PRC (15 iterations and 12 subsets) and (**b**) Small-tumor phantom with OSEM (2 iterations and 12 subsets), OSEM+PRC simplified (2 iterations and 12 subsets), OSEM+PRC (9 iterations and 12 subsets), PSF (8 iterations and 12 subsets), PSF+PRC simplified (7 iterations and 12 subsets) and PSF+PRC (18 iterations and 12 subsets). For the NEMA IQ phantom, the highest differences (%) seen were for the smaller spheres 10, 13, and 17 mm and the edge of the phantom

The visual inspection of the reconstructed NEMA IQ phantoms is shown in Fig. 2. With OSEM+PRC and PSF-PRC simplified, Gibbs artifacts were introduced, which became more pronounced for PSF+PRC (Fig. 3). Regardless of the PRC implementation, the effect was more pronounced for the smaller spheres (10, 13, and 17 mm).

Table 1 summarizes the lesion contrasts with all PRC implementations for a clinically acceptable image noise of ~ 10%. For subsequent evaluations, those reconstruction settings were selected that yielded a background noise level of ~ 10% following the convergence analyses (Fig. 1).

**Table 1:** Recovery coefficient for the different image reconstruction and kernel combinations. The image reconstruction settings were defined to match the background noise around ~ 10% for the NEMA IQ phantom and ~ 33% for the Small-tumor phantom. Relative deviations (%) apply relative to the OSEM and for PSF reconstructions

	Recovery coefficient (relative difference [%])						Background noise [%] (relative difference [%])
Sphere size	10 mm	13 mm	17 mm	22 mm	28 mm	37 mm	
OSEM	0.39	0.50	0.64	0.75	0.77	0.85	9.73
OSEM+PRC simplified	0.43 (9.9)	0.56 (11.3)	0.71 (10.8)	0.79 (5.4)	0.82 (4.7)	0.87 (2.4)	10.83 (11.4)
OSEM+PRC	0.53 (36.5)	0.63 (25.0)	0.73 (15.4)	0.81 (8.2)	0.83 (7.8)	0.88 (4.4)	9.35 (−3.8)
PSF	0.56	0.64	0.75	0.83	0.85	0.89	9.32
PSF+PRC simplified	0.66 (18.7)	0.74 (15.7)	0.85 (13.4)	0.87 (5.1)	0.89 (5.3)	0.92 (3.6)	9.82 (5.3)
PSF+PRC	0.64 (15.7)	0.72 (12.0)	0.81 (8.2)	0.85 (3.1)	0.88 (3.7)	0.92 (2.8)	9.78 (4.9)

Sphere size	3.7 mm	4.8 mm	6.5 mm	7.7 mm	8.9 mm	9.7 mm	
OSEM	0.09	0.16	0.28	0.31	0.36	0.44	32.9
OSEM+PRC simplified	0.10 (6.5)	0.20 (23.5)	0.39 (40.0)	0.45 (42.3)	0.49 (35.1)	0.60 (35.4)	37.5 (13.9)
OSEM+PRC	0.15 (71.9)	0.29 (80.4)	0.52 (85.9)	0.54 (73.2)	0.58 (60.8)	0.66 (49.6)	33.5 (1.7)
PSF	0.15	0.27	0.47	0.50	0.54	0.62	32.10
PSF+PRC simplified	0.17 (14.7)	0.34 (27.3)	0.70 (47.6)	0.73 (46.2)	0.76 (41.0)	0.86 (39.8)	33.4 (4.1)
PSF+PRC	0.20 (36.6)	0.36 (35.1)	0.70 (48.2)	0.75 (49.0)	0.79 (45.3)	0.87 (41.1)	33.9 (5.6)

### Small-tumor phantom

**Fig. 4:**
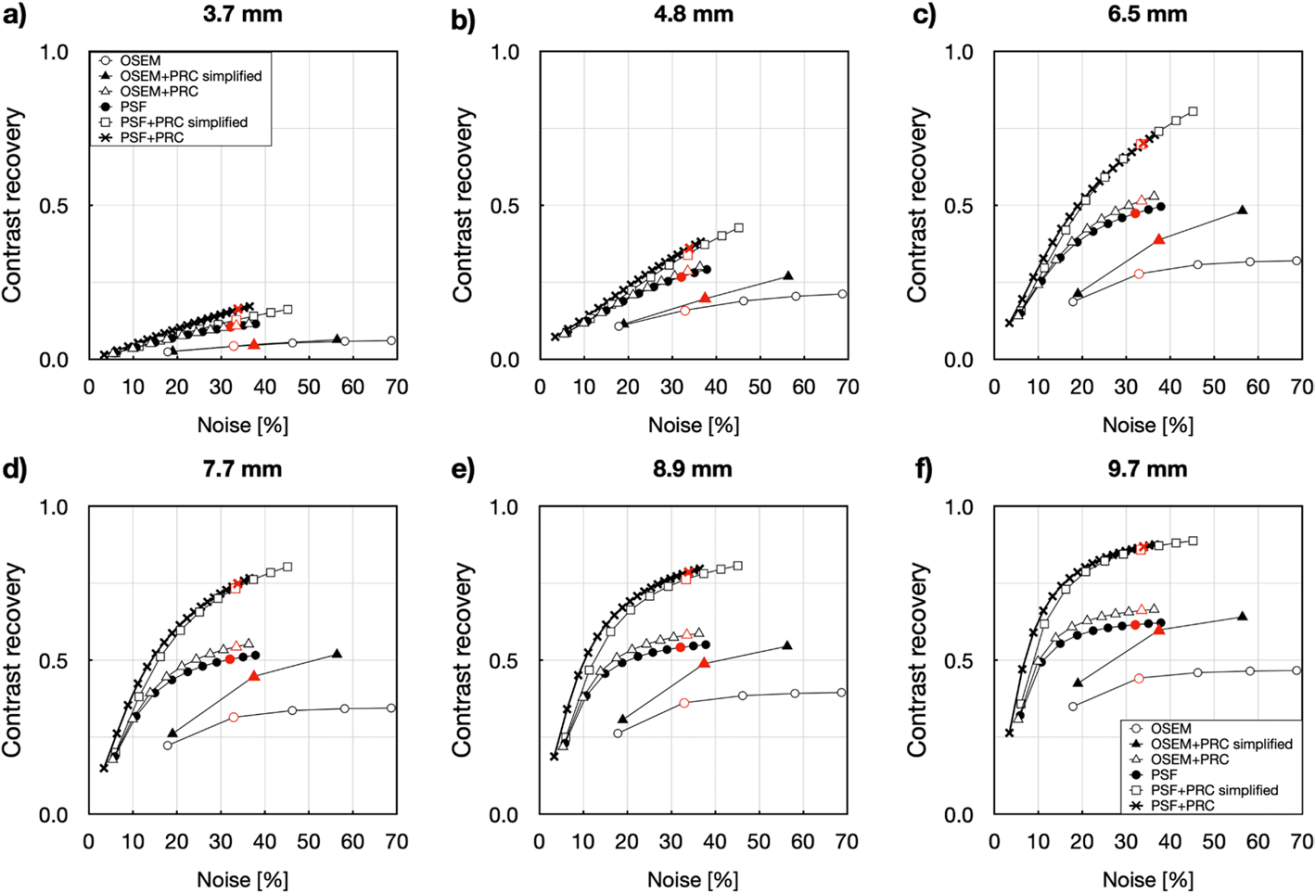
Noise vs. contrast recovery for the Small-tumor phantom (**a**) 3.7 mm, (**b**) 4.8 mm, (**c**) 6.5 mm, (**d**) 7.7 mm, (**e**) 8.9 mm, 9.7 mm spheres filled with ^124^I and reconstructed with OSEM (1–5 iterations), OSEM+PRC simplified (1–3 iterations), OSEM+PRC (1–10 iterations), PSF (1–10 iterations), PSF+PRC simplified (1–10 iterations), and PSF+PRC (1–20 iterations). Every point corresponds to one iteration (all reconstructions done with 12 subsets). The behavior of the OSEM+PRC is similar to the stand-alone PSF reconstructions. The selected number of iterations for the comparison of the images are marked with red

For this phantom, similar convergence patterns were observed as in the case of the NEMA IQ phantom (Fig. 4). The effect of PRC was noticeable for hot lesions down to 4.8 mm. When comparing images with similar noise levels (~ 33%) (Fig. 2), OSEM+PRC improved contrast recovery by 80% and 86% for the 4.8 and 6.5 mm spheres, respectively (Table 1), which is also clearly seen visually (Fig. 3b). Table 1 summarizes the contrasts improvements with all PRC implementations for a comparable image noise level of ~ 33%.

### Bone–lung phantom

**Fig. 5:**
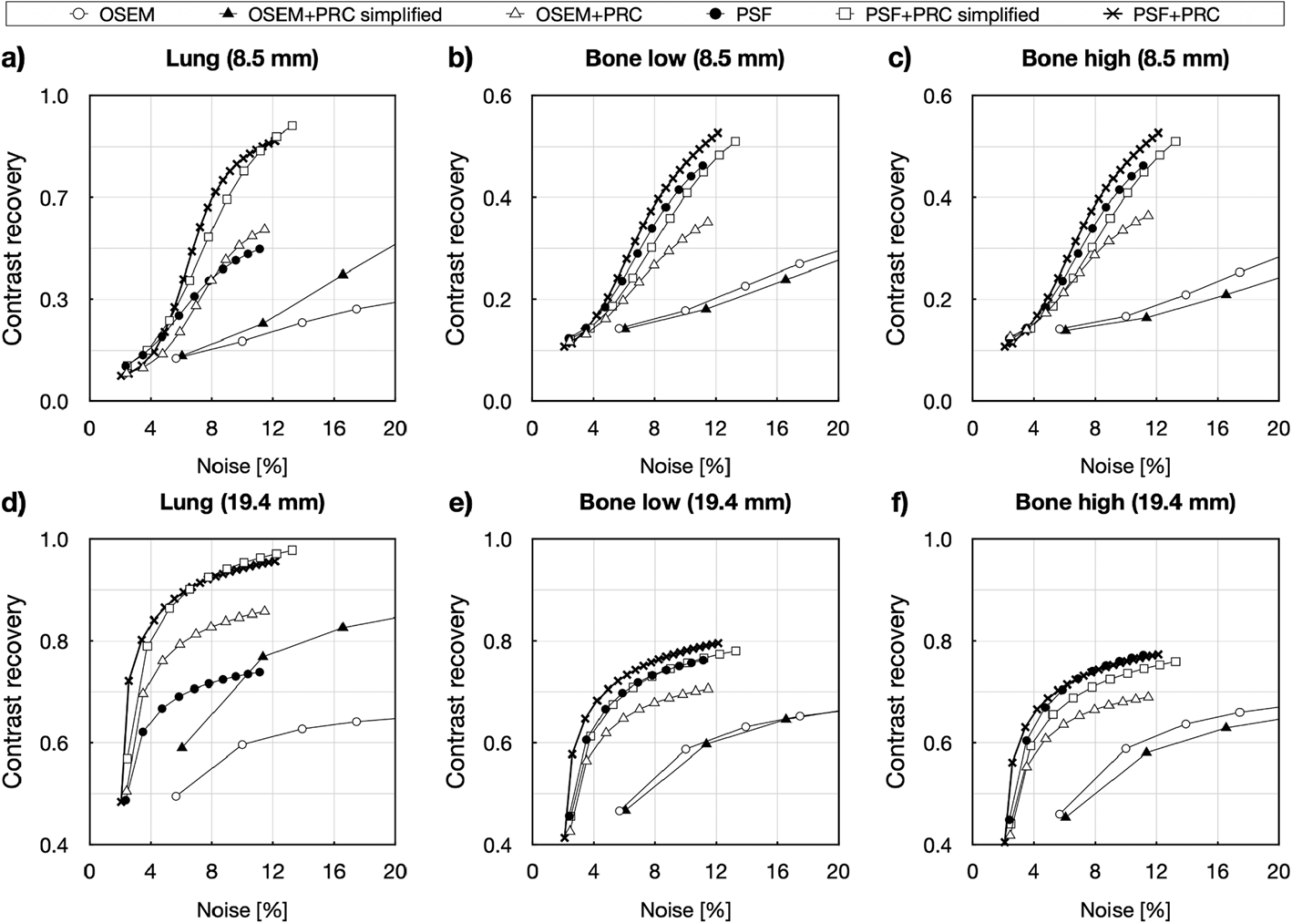
Contrast recovery vs. noise calculated for the small sphere (8.5 mm) in (**a**) lung, (**b**) bone (500 HU), (**c**) bone (1000 HU) and for the large sphere (19.4 mm) in (**d**) lung, (**e**) bone (500 HU), (**f**) bone (1000 HU) reconstructed with PRC methods in combination with OSEM and PSF. The changes are most pronounced within the lung insert

**Fig. 6:**
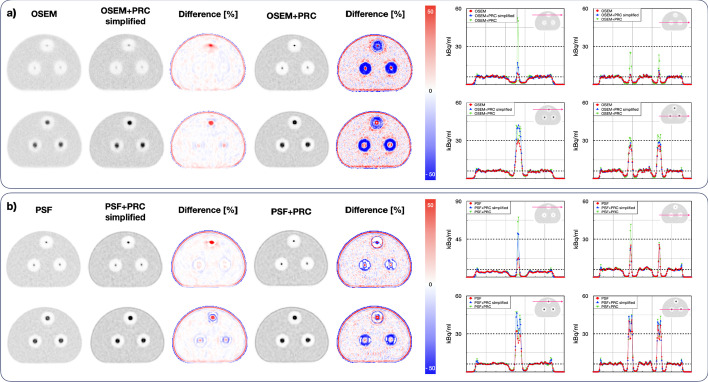
Comparison of the reconstructed Bone–lung phantom with (**a**) OSEM and (**b**) PSF the central slices are shown through the small (8.5 mm) and (**b**) the central slices are shown through large (19.4 mm) spheres. The recovery is slightly overestimated for the 8.5 mm sphere within the lung medium. The black dashed lines represent the activity concentrations in the hot spheres and background

The convergence of the reconstructions was highly affected by the surrounding medium. Within the bone inserts, almost no change in contrast improvement was achieved for either the small or big spheres with the OSEM+PRC simplified (Fig. 5). The convergence was similar for OSEM+PRC, PSF, and PSF+PRC reconstructions. Within the lung medium, the effect of PRC was clearly visible with substantially improved contrasts (Table 2). The OSEM+PRC simplified showed similar convergence as OSEM with a substantially higher contrast recovery. The trend was similar with OSEM+PRC and PSF reconstruction with reduced image noise. PSF+PRC simplified and PSF+PRC had almost identical convergence, with the simplified implementation leading to convergence substantially faster. The effect of PRC within the lung medium is also clearly visible on the difference images shown in Fig. 6. Table 2 summarizes the contrast recoveries for all reconstruction and PRC combinations at comparable image noise (~ 10%).

**Table 2:** Contrast recovery for every reconstruction method and kernel variation using the Bone–lung phantom. Relative deviations (%) in relation to the standard OSEM and for th﻿e PSF reconstructions

Sphere size	8.5 mm			19.4 mm		
Material	Lung	Bone	Bone	Lung	Bone	Bone
	(−800 HU)	(500 HU)	(1000 HU)	(−800 HU)	(500 HU)	(1000 HU)
OSEM	0.20	0.18	0.17	0.60	0.59	0.59
OSEM+PRC simplified	0.25 (29.4)	0.18 (1.6)	0.16 (−1.7)	0.77 (29.0)	0.60 (1.7)	0.58 (−1.4)
OSEM+PRC	0.57 (190.5)	0.33 (88.7)	0.36 (114.7)	0.85 (41.9)	0.70 (18.4)	0.68 (15.7)
PSF	0.43	0.38	0.35	0.72	0.74	0.75
PSF+PRC simplified	0.66 (52.8)	0.36 (−5.8)	0.31 (−12.3)	0.94 (30.1)	0.74 (0.4)	0.72 (−3.6)
PSF+PRC	0.79 (83.8)	0.47 (23.0)	0.52 (48.5)	0.94 (30.3)	0.78 (5.2)	0.76 (1.1)

## Discussion

In this paper, different PRC implementation methods in combination with PSF image reconstruction were evaluated for PET imaging using ^124^I. The implementation of the PR-dependent blurring into the PET image reconstruction was done for the OSEM algorithm and for a PSF corrected OSEM as full and simplified implementation [14]. Both PRC implementations, simplified and full implementation, lead to increased contrast recovery compared to standard OSEM. However, the full implementations also led to a slower convergence of the algorithm although with higher achievable contrasts compared to with PRC simplified. The simplified implementation demonstrated a faster convergence although with substantially higher noise levels (Fig. 1). The reason for the faster convergence and the increased noise levels are expected due to result from the strong mismatch of the forward and backward projectors [3].

The image reconstruction with OSEM+PRC showed a similar convergence to the PSF reconstruction (Fig. 1). This can be explained by the similarity of the full implementation of the PRC and the vendor-based PSF image reconstructions [3].

When adding the PRC to the PSF reconstruction, the image contrast recovery was almost identical for the full and the simplified PRC implementation. This observation may be explained by the higher similarity of the forward and backward projector in the case of the combination of the PRC with PSF. This observation is also strengthened by the results for the OSEM-PRC simplified, which indicates that the mismatch of forward and backward projector substantially influences the noise propagation within iterative PRC implementations (Figs. 2 and 3).

The effect of PRC was the most pronounced within low-density materials, such as lung, where the reconstructions with PRC outperformed the standard reconstructions (Fig. 4). In this case, also OSEM+PRC was leading to higher contrast recovery compared to PSF (Fig. 5). On the other hand, within the bone medium, relatively small changes were seen for PRC-based reconstructions. This was expected due to the high positron range of ^124^I within the lung (max. 30 mm) and short PR in bone. Gibbs artifacts well known from PSF reconstructions were also present in the PRC reconstructions and more prominent when PSF and PRC were combined (Figs. 3 and 6). As shown in Fig. 6 in the line profile through the small spheres, a slight overestimation can be caused by the Gibbs artifacts. The effect is more visible in the edges of the bigger spheres. However, such Gibbs artifacts as known from PSF are mainly occurring at sharp activity concentration borders as present in phantom studies and are expected to be less pronounced in patients. Further, as shown for PSF reconstructions, such artifacts can be handled using post-reconstruction filters or by applying regularization methods [25, 26].

## Limitation

The composition of the spatially variant and tissue-dependent kernels does not take into account the positron energy loss, in particular when a positron is emitted from a higher density material. The solution for these limitations would require a more complex PR such as based on direct MC simulations; however, due to an extensive number of kernels that have to be computed during the image reconstruction process, the full PRC application is still a challenge especially with the aim of clinical application.

The data acquisition of the NEMA IQ phantom was 60 min. For obtaining similar count statistic as for a standard 15 min NEMA IQ scan with ^18^F given the reduced branching ratio for positrons in ^124^I. In the clinical scenario, the reconstruction settings may have to be adjusted to account for the reduced count statistics due to the shorter acquisition times.

The evaluated PRC methods are based on the same basic methodology of calculating the spatially variant and tissue-dependent kernels, by analyzing the underlying material composition from the AC maps [14]. Thus, the errors in the attenuation map directly translate into artifacts caused by the PRC. This holds also true in the case of PET/MRI, were artifacts in MR-based AC might occur more frequent that for PET/CT. Furthermore, when applying the evaluated methods for the PET/MRI, the effect of the magnetic field on the PR has to be carefully considered [15, 27].

## Conclusion

Combining PSF and PRC notably increases achievable contrast compared to PSF or PRC alone in ^124^I PET and appears to be preferred for formal correctness, contrast recovery, and noise level. Our study showed that the use of a simplified PRC implementation applying the PR-based blurring exclusively to the forward projector within an OSEM algorithm, resulted in a seriously increase in image noise compared to the full implementation of PRC in both, forward- and back-projection. However, a simplified implementation of PRC in the forward projector appears acceptable, when combined with a PSF correction.

## Supplementary Information

**Additional file 1**. Employed phantoms: a) NEMA IQ - Image quality phantom; b) Small tumor phantom and c) Bone-lung phantom. d-f) VOI delineations for the image analysis.

## Data Availability

The datasets generated and/or analyzed during the current study are available from the corresponding author on reasonable request.
