# Supplementary material for: Positron range in combination with point-spread-function correction: an evaluation of different implementations for [124I]-PET imaging
Source: EJNMMI Phys. 2022 Aug 19;9:56. doi: 10.1186/s40658-022-00482-y (PMC9391565; doi:10.1186/s40658-022-00482-y)
Supplement: Supplementary file 1 — Additional file 1. Employed phantoms: a) NEMA IQ - Image quality phantom; b) Small tumor phantom and c) Bone-lung phantom. d-f) VOI delineations for the image analysis. [file 40658_2022_482_MOESM1_ESM.docx]

**SUPPLEMENTARY FIGURE**


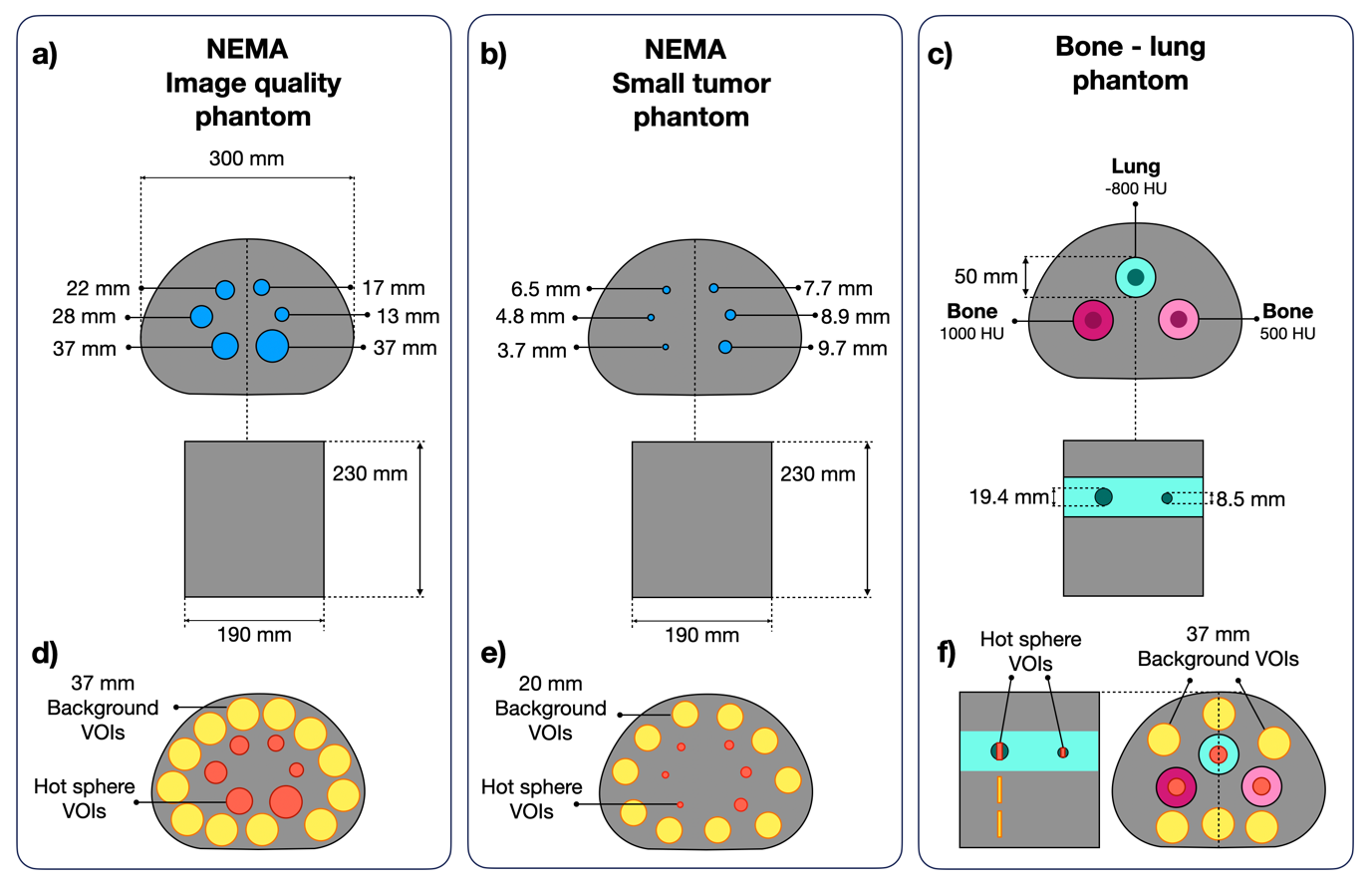


**Supplementary Figure 1.** Employed phantoms: a) NEMA IQ - Image quality phantom; b) Small tumor phantom and c) Bone-lung phantom. d)-f) VOI delineations for the image analysis.

**
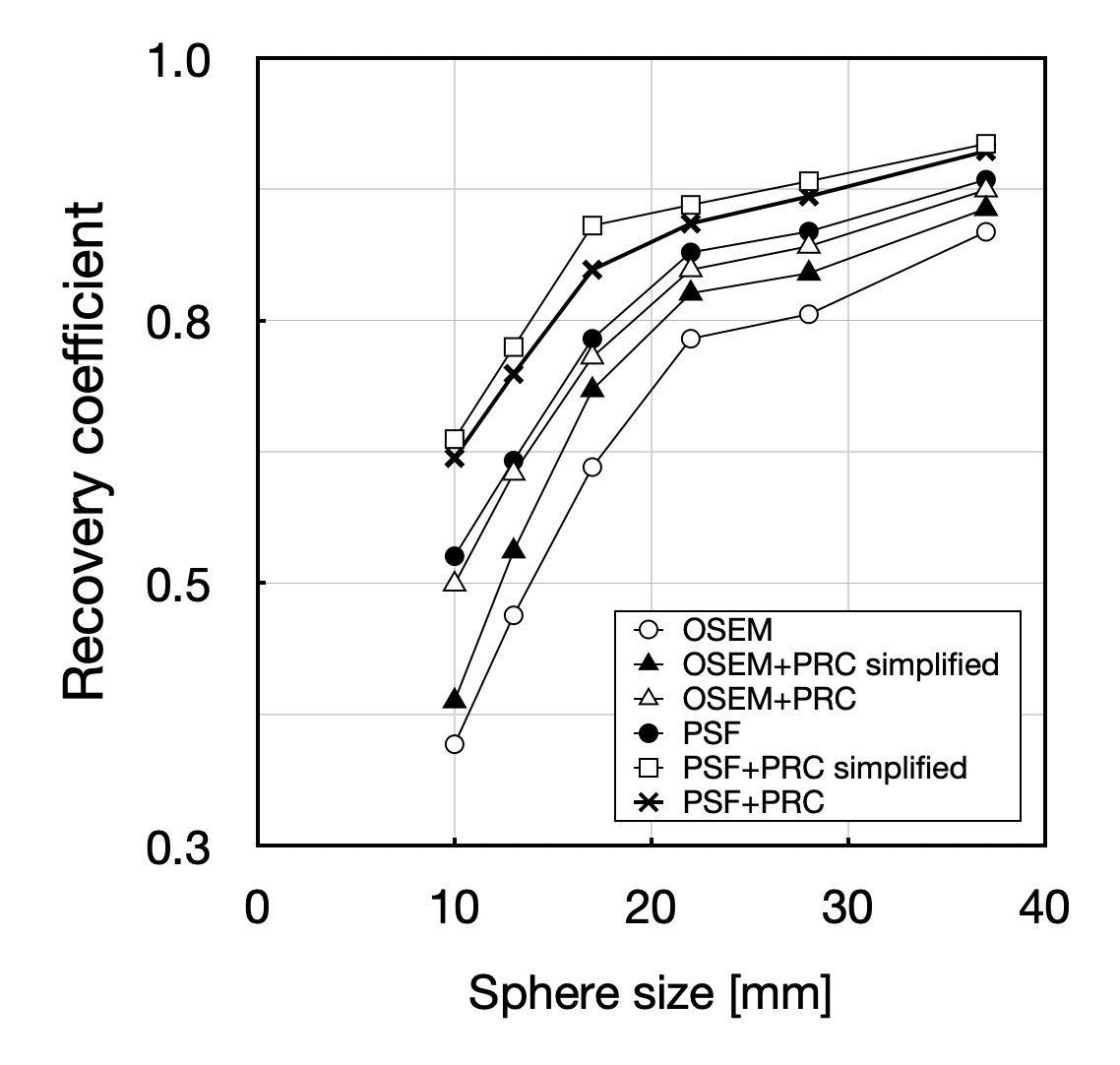
**

**Supplementary Figure 2.** Sphere size vs. Recovery coefficient for the NEMA IQ phantom for all image reconstruction and PRC variations.
